# Polio-Like Manifestation of Powassan Virus Infection with Anterior Horn Cell Involvement, Canada

**DOI:** 10.3201/eid2508.190399

**Published:** 2019-08

**Authors:** Christopher Picheca, Vignan Yogendrakumar, James I. Brooks, Carlos Torres, Elizabeth Pringle, Jocelyn Zwicker

**Affiliations:** University of Ottawa, Ottawa, Ontario, Canada

**Keywords:** anterior horn cells, arboviruses, magnetic resonance imaging, encephalitis viruses, tick-borne, poliomyelitis, Powassan virus, vector-borne infections, Canada, viruses, meningitis/encephalitis

## Abstract

Evidence of spinal cord involvement in Powassan virus infection is largely limited to mouse models. We report a case of a polio-like illness caused by Powassan virus infection in a 62-year-old man in Canada. Magnetic resonance imaging showed T2 hyperintensities in the anterior horns of the cervical spinal cord.

Powassan virus (POWV) is a tickborne flavivirus, named after Powassan, Ontario, Canada, the location of the first documented human infection in 1958 (*1*). Since then, ≈150 cases of POWV infection have been reported globally, and incidence has increased over time. A total of 125 POWV cases have been identified since 2008, 33 (26%) in 2017 (*2*). In Canada, most reported POWV infections have been in the Great Lakes region. A small number of cases have been reported in the Maritime provinces (*3*). 

POWV is transmitted by members of the *Ixodes* genus of ticks, including *I. cookei* and the more opportunistic and aggressive *I. scapularis*. POWV has 2 lineages; lineage 2 (deer tick virus) has emerged quickly in parts of North America, along with the expanding range of *I. scapularis* ticks.

POWV infection typically begins with prodromal symptoms including fever, nausea, headache, and myalgia. Central nervous system involvement includes an altered level of consciousness, paralysis, or ophthalmoplegia (*4*). POWV encephalitis has a 10% mortality rate, and <50% of survivors suffer residual deficits (*5*). Studies with mice have demonstrated that POWV can affect motor neurons in the anterior horns of the spinal cord (*6*). These same neurons are affected by poliovirus, West Nile virus, and enterovirus D68 (*7*). However, POWV infection with cord involvement in humans is not well documented; 1 human case demonstrated motor neuron pathology after POWV lineage 2 infection (*8*), and a second case with suspected motor neuronopathy was reported in 2018 (*9*).

We present the case of a 62-year-old man living in urban Ontario who experienced nausea, vomiting, and abdominal pain while vacationing in rural Newfoundland. He sought treatment at a hospital in Nova Scotia and experienced diplopia and ataxia. A computed tomography scan of the head did not show any acute intracranial event.

The patient became febrile and experienced dysarthria, weakness, and respiratory distress. Cerebrospinal fluid analysis showed pleocytosis (159 × 10^6^ total nucleated cells: 42% neutrophils, 43% lymphocytes) and elevated protein levels (0.79 g/L). He was started on empiric treatment with ceftriaxone, ampicillin, acyclovir, and dexamethasone. Results of tests for *Cryptococcus*, HIV, syphilis, Lyme disease, herpes simplex viruses 1 and 2, varicella zoster virus, and acid-fast bacilli were negative. Initial arbovirus serology results were negative. The patient worsened, requiring intubation and transfer to an intensive care unit.

Seven days after arriving at the hospital, the patient was unable to lift his arms and was transferred to a tertiary center in Ottawa, Ontario (The Ottawa Hospital). Neurologic exam showed facial and extraocular muscle weakness. He had flaccid tone and absent power in his upper extremities and reduced strength in his lower extremities. Sensation was preserved. Nerve conduction studies demonstrated diffusely low motor amplitudes, normal sensory amplitudes, and normal conduction velocities suggestive of a motor neuronopathy. Electromyography in the acute phase was not possible due to poor patient cooperation.

Results of paired convalescent arbovirus serology collected 1 month after symptom onset were positive. Testing at the National Microbiology Laboratory confirmed POWV infection (hemagglutination inhibition titer 1:80, plaque-reduction neutralization titer 1:160). Magnetic resonance imaging (MRI) of the brain showed infratentorial and supratentorial leptomeningeal enhancement. An MRI of the cervical spine showed increased T2-weighted signal involving the anterior horns from C3 to C6 (Figure; Appendix Figure).

**Figure:**
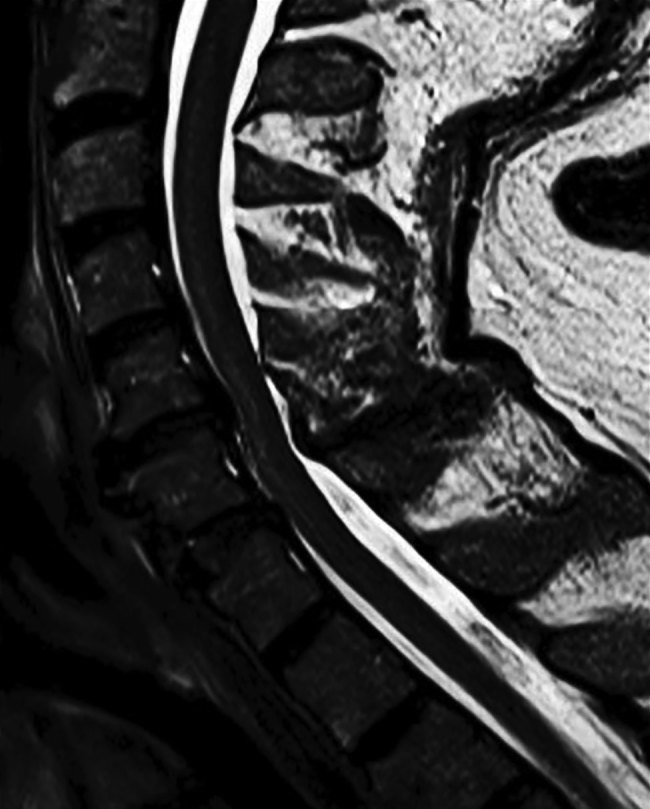
Sagittal T2-weighted image of cervical spinal cord in a patient with Powassan virus infection, Canada. A longitudinal hyperintensity of the anterior horn is visible from C3 to C6.

Follow-up MRI of the brain and spine 1 month later showed interval resolution of leptomeningeal abnormalities, but abnormal signal within the anterior horn of the cervical spine remained. Electrodiagnostic testing repeated 6 months after symptom onset again showed normal sensory nerve conduction studies and abnormal motor nerve conduction studies. There was diffuse denervation in all cervical myotomes including the paraspinal muscles, confirming a motor neuronopathy consistent with a poliomyelitis-like presentation of POWV. We suspect that initial arbovirus serology was performed too early in the disease course, because paired serology 4 weeks later demonstrated seroconversion and confirmed diagnosis.

Our case shares similarities with a recently published report of POWV infection (*9*). In that case, a patient vacationing in the Luskville region of Quebec, Canada, experienced cranial nerve pathologies and flaccid weakness of the upper extremities. Electrodiagnostic testing showed evidence of diffuse denervation and reinervation across multiple myotomes that was consistent with a motor neuronopathy. Imaging of the brain showed only mild hyperintensities that would not account for the patient’s disproportionate weakness. A spinal MRI was not performed. As in our case, acute arbovirus serology results were initially negative but were positive in paired testing (*9*).

The incidence of POWV infection has increased since 2017 (*7*). *I. scapularis* ticks and deer tick virus may be becoming more widely disseminated in northern regions, potentially due to warming climates, whereas infection with prototypical POWV transmitted by *I. cookei* ticks remains rare and stable in distribution (*10*). It is possible that Canada will see an increasing number of cases similar to ours. Our findings emphasize the need to include POWV infection in the differential diagnosis for patients with polio-like symptoms in tick-endemic regions.

Appendix Additional information about poliomyelitis-like presentation of Powassan virus with anterior horn cell involvement, Canada.
