# Supplementary material for: Polio-Like Manifestation of Powassan Virus Infection with Anterior Horn Cell Involvement, Canada
Source: Emerg Infect Dis. 2019 Aug;25(8):1609–11. doi: 10.3201/eid2508.190399 (PMC6649312; doi:10.3201/eid2508.190399)
Supplement: Appendix — Additional information about poliomyelitis-like presentation of Powassan virus with anterior horn cell involvement, Canada. [file 19-0399-Techapp-s1.pdf]

Article DOI: https://doi.org/10.3201/eid2508.190399

Polio-like Manifestation of Powassan Virus Infection with Anterior Horn Cell Involvement, Canada

Appendix

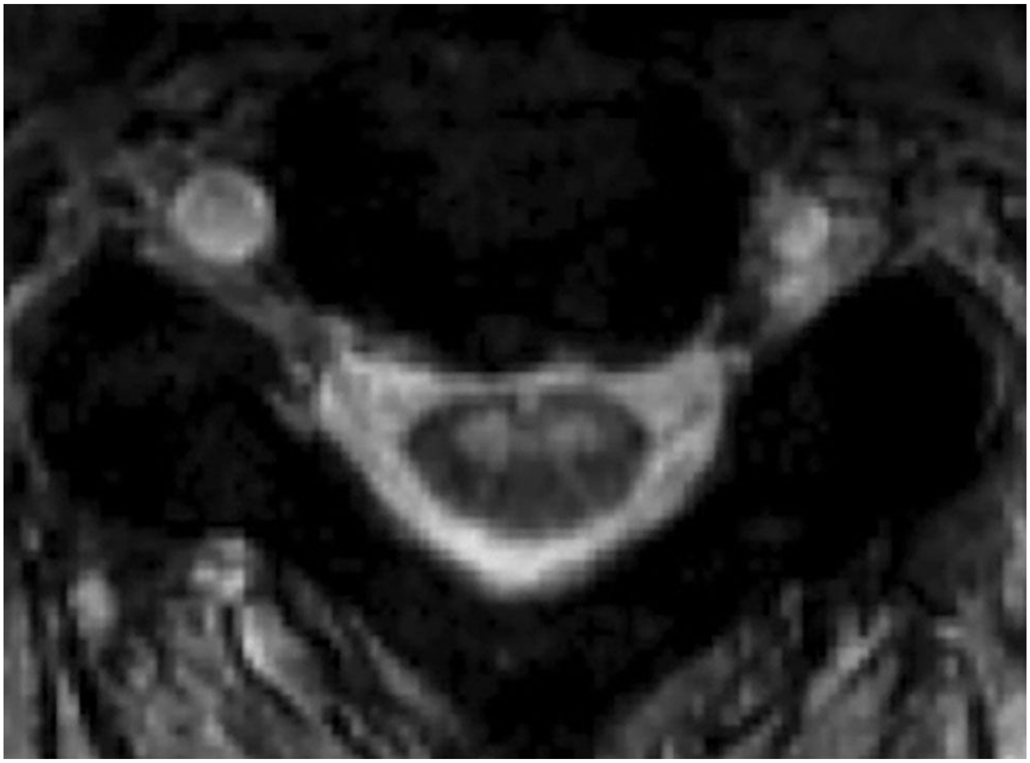

Appendix Figure. Axial T2-weighted image of cervical spinal cord in a patient with Powassan virus infection. A longitudinal hyperintensity of the anterior horn is visible from C3 to C6.
